# Data disaggregation: the case of Asian and Pacific Islander data and the role of health sciences librarians

**DOI:** 10.5195/jmla.2022.1372

**Published:** 2022-01-01

**Authors:** Seema Bhakta

**Affiliations:** 1 seema.bhakta@providence.org, Medical Librarian, System Library Services, Providence Health & Services, Portland, OR

**Keywords:** data disaggregation, Asian and Pacific Islander, health sciences librarians, health sciences librarianship

## Abstract

Health disparities within Asian and Pacific Islander (API) communities are often masked due to aggregated data. Lack of adequate data limits required health care services for these communities. While moving forward toward health equity, it is critical that disparities for API communities are acknowledged and addressed. This article focuses on the issues of aggregated data for API communities followed by suggestions on how health sciences librarians can support and promote better practices for data disaggregation.

## INTRODUCTION

Information about existing health disparities between different racial and/or ethnic groups is often presented with tables and graphs to unfold themes of social determinants of health. However, visual representation of data frequently shows rates of acute or chronic diseases in Asian and Pacific Islander (API) communities to be low or nonexistent compared to other racial and/or ethnic minority groups. This observation leads to the assumption that there are no existing disparities for this population and limits required health care services for API communities. Asia consists of over forty countries, and the Pacific Islands are grouped by three subregions of Oceania (including Native Hawaiians); both have a diaspora spread across the globe. Due to differences in social, economic, and environmental factors, it is erroneous to assume that health disparities for this population do not exist. Thus, it is essential to disaggregate demographic data to reveal disparities among the API communities. The practice of data disaggregation is to break up collected data into separate subgroups to highlight specific underlying patterns. For example, data collected for API can be separated into different ethnic subgroups such as Chinese, Japanese, Korean, etc. or Samoan, Tokelauan, Tahitian, Tongan, etc.

Data for API communities, including immigrants and refugees, are frequently combined, masking heterogeneity within the group [1]. Aggregated data hide the unique and complex health care needs of API communities by overshadowing sociocultural and sociodemographic factors such as variation in immigration and refugee experiences, income and poverty levels, history of discrimination, and process of acculturation [2]. For example, data inequity has become visible during the recent COVID-19 pandemic, and although states are required to report disaggregated data, the rates for Native Hawaiians and Pacific Islanders (NHPI) have not been acknowledged in media reports [3]. An analysis of California's COVID-19 data shows the challenges of aggregated data, as it highlights a smaller risk for NHPI when data are grouped together with Asian communities, when in fact the risk is higher than the overall state population [4]. In 2020, California's crude mortality rate for COVID-19 was 84 per 100,000 population, the crude mortality rate for Asian and NHPI combined was 75 per 100,000, and the crude mortality rate for NHPI alone was 123 per 100,000 [4]. Therefore, when data are aggregated, disparities for a specific community within the API group will be unknown, and these differences are important to acknowledge in terms of developing preventive and screening efforts for specific subgroups [1]. For example, aggregated data from electronic health records (EHRs) underestimates the prevalence of diabetes for Filipinos, South Asians, and Pacific Islanders [5]. Therefore, these subgroups may be overlooked for early screening and/or treatment.

Data disaggregation ensures that the unique issues facing specific API communities are addressed and thus requires accurate and detailed data collection [6]. For example, in order to calculate incidence and prevalence of disease, it is important to have population-level data. However, while many national surveys and registries are an important source for population data, prior to 2003, they did not collect data by racial subgroups, thus aggregating the data [1]. Aggregated data are a problem because data from comprehensive federal surveys are routinely used by researchers and policymakers to set the agenda of needed services [1].

Although it is possible to study an entire population, many researchers sample participants drawn from the population, which requires adequate sampling techniques to ensure equal representation within the sample [7]. By adequately sampling subgroups, disaggregation can provide meaningful and critical information on the health needs of specific ethnic populations to community service providers and policymakers [1,2]. It is also essential to have a large sample size to make it less likely that the participants will differ significantly from the population of interest. Smaller samples of API subgroups lead to statistically unreliable estimates, suggesting that differences do not exist, when in fact larger, more adequate samples will reveal important differences [1], such as for self-reported diabetes prevalence [8], patient-provider communication [9], and the risk of gestational diabetes [10].

Inadequate data collection methods impact the reliability and validity of the data gathered and leads to misinterpretation of data [1,2]. However, data collection for API subgroups is challenging because it is difficult to recruit API participants for studies, which has often been attributed to mistrust, lack of information, and language and cultural barriers [1]. While there are national, state, and community efforts to collect subgroup data, it is important to offer interviews and surveys in multiple languages and dialects that span the API diaspora. However, API languages are generally not used in most studies, and sampling methods may omit participants with limited English proficiency and leave them out from interviews because of the language barriers [1]. Sampling bias occurs and data are skewed when only English-speaking participants are interviewed [1,2]. Self-identification also plays an important factor and can result in undercounting or missing data due to issues of ethnic classification. It is important to understand how social differences or sociopolitical history impacts ethnic identification or opting out of an identity. For example, Punjabis in Northern California have been found to not identify as Asians because of their sociopolitical history in India, and South Asians living in the United States predominantly identify as Indian-American [2,11].

## THE ROLE OF THE HEALTH SCIENCES LIBRARIAN

Advancing health equity and reducing health disparities requires better practices for research design and data collection. The lack of systematic disaggregation of data, whether it be at the collection, analysis, or reporting phase, can disguise essential health care needs, social services, and resources for the API communities [3]. Differences between the API subgroups are important to acknowledge in terms of developing targeted preventive and screening efforts and requires collaboration to implement new practices to change the data system [1,3].

Health information professionals serve a variety of patrons to meet their information needs and go beyond their exclusive role as information providers to take on more active roles such as public health collaborators, research experts, and/or data managers. The following sections offer suggestions and are not exhaustive of what librarians can do, but allow us to begin thinking about our role when it comes to addressing the challenges of aggregated data and promoting data disaggregation practices.

### Outreach & advocacy

Health sciences research is neither inclusive nor accessible to different demographics. Minorities are continuously underrepresented in clinical trials and epidemiologic cohort studies as there are barriers to information, limited understanding due to language barriers, low community engagement, and lack of trust and awareness, all of which limit opportunities to participate in studies [12,13]. For example, when recruiting for clinical trials, it is vital that there is diverse representation of the population [13,14]. As recruitment has been relatively homogenous, proper outreach is needed to have a heterogenous study population [14,15].

The National Institutes of Health (NIH)'s All of Us program is working to improve health care through research and focuses on building a diverse database by recruiting a diverse participant pool that includes members of groups that are often left out in research [16]. The NIH/Network of the National Library of Medicine (NNLM) All of Us Community Engagement Network provides awareness of the All of Us program to underrepresented communities [17]. The current All of Us program data groups Asian as a single unit, possibly because there is low recruitment and representation, thus requiring more outreach in these communities. As members of the NNLM, health sciences and medical libraries can access and distribute materials about the program to their patrons. Librarians can also partner with other programs like All of Us and/or research centers within their institutions to offer services and strategies on outreach and recruitment.

A key aspect of research recruitment and participation is informed consent. Trustworthiness is an essential component to the widespread implementation of how data are collected, how data are used, and who has access to data [18]. The principle of informed consent requires that participants are provided with information about the project that is sufficiently full and accessible in order to be considered informed for their decision about whether to take part [19]. Informed consent establishes a more equal relationship between researchers and participants, in which the latter can have the confidence to be more open and frank about the aspects of their lives that are being researched, resulting in collection of quality data [19].

Participants from API communities are more likely to participate in studies when there are culturally matched researchers, language-appropriate tools and resources, and community involvement to destigmatize participation in research and altruism [20]. For example, the Pacific Islander community has concerns related to the use of data extrapolation that may overgeneralize Native Hawaiians, the need for community input within the scope of the research, and the requirement for culturally tailored programming that is directed back to the community [20]. Partnership with API community organizations is important for recruiting participants and decreasing information inequities by addressing questions and concerns. Librarians can partner with API organizations and/or work with translators to develop appropriate resources in API languages and dialects for outreach and recruitment, to provide information addressing questions and concerns about research projects, and to ensure community representation of participants with limited English proficiency and/or low literacy [1]. For example, some libraries have partnered with researchers and organizations to understand information-seeking behavior of different communities and to develop resources with community health workers (CHWs) [21,22]. As members of the community they serve, CHWs have a unique understanding of the norms, attitudes, values, and strengths of their community and can bridge the information and communication gap between members and health institutions [23]. Although CHW programs do not adequately address the ethnic and cultural diversity in API populations or address the causes of morbidity and mortality in subgroups, CHWs have a unique position to influence social connectedness, social capital, and social support within these communities [23]. Thus, the library and CHW partnership model can be applied to API communities to bridge the gap of subgroup outreach for research recruitment and understanding of health information needs, information-seeking behavior, and health literacy. While CHWs are effective in community outreach, there is a lack of integration within clinical settings [23]. Librarians, especially those interested in consumer health librarianship, can advocate for CHW recruitment in clinical settings as part of consumer health resource-sharing initiatives. Partnerships with CHWs and librarians would also be a valuable model of engagement with communities through community-based participatory research efforts to understand and utilize best practices of outreach in underserved community-driven populations such as Pacific Islander communities [24].

### Data ethics & services

It is essential for librarians to acknowledge the importance of data disaggregation in the instruction of data literacy and data equity. Through curation, preservation, and dissemination of data, librarians can also develop ethical activities in partnership with researchers, students, faculty, and clinicians to advance disaggregated practices across the life cycle of the data [3]. For example, EHRs are used to gather and display patient-level data, like race and ethnicity, for clinical decision-making. However, during initial patient visits, data on race and ethnicity are not often captured; although modifications are made over time, there is variance in completeness of information [25]. The accuracy of race and ethnicity reported data in EHRs is also limited and requires the need for a better process of documentation [26], adjusting the EHR record system by offering additional options, and providing multidisciplinary education efforts for clinicians and patients [27]. Librarians can highlight the importance of documenting race and ethnic subgroups in EHRs to residents, faculty, and clinicians. Advocating for subgroup documentation would allow librarians to provide appropriate information resources for better patient care.

Data ethics are tied to societal contexts and understanding, and working with communities that have been previously marginalized, underrepresented, and misrepresented includes principles of privacy, research ethics, ethical ecosystems, and control [28]. Ethical activities ensure individual privacy and confidentiality of data; maintain integrity of research through the protection of data and prevents data manipulation; safeguard spaces, communities, cohorts, and other information ecosystems where data are created, accessed, and studied; and guide the decisions regarding who is in control of data, who should be in control, and how data are preserved and accessed [28].

Privacy is the “claim of individuals … to determine for themselves when, how and to what extent information about them is communicated” [29], which is a central concern within the library and information science (LIS) field [28]. Since data is managed within different information ecosystems such as medical and/or academic sectors, librarians can enforce an ethical framework of checks and balances among each user of data [28]. Within LIS, attention on data as intellectual property and academic integrity is a key focus within research ethics. As librarians work with faculty, students, and clinicians on research and scholarly communication, it is important to be aware of plagiarism, faking of data, unethical data collection methods, archiving sensitive data, and informed consent [28]. Value of data is based on the ability to create governing structures and delivery of better policies and strategies [30].

Data repositories have been created in many disciplines to preserve data to promote wider sharing and reuse of data [31]. Librarians have the unique qualification to manage and control data repositories, such as overseeing who can use the data and how the data are used while keeping in mind the principles of information privacy and confidentiality [31]. Governing access and use are important for data with policy, legal, or ethical considerations [31]. For example, data collected from interviewing immigrants, refugees, and those who are undocumented are delicate but necessary for disaggregated data; therefore, it is important to keep the data private, confidential, and deidentified [2]. Privacy is a major concern in research involving human subject data; thus, participants' informed consent must be respected and data deidentified for data sharing [31]. Controlled data collections are repositories in which librarians can make and enforce rules with data sharers and data users for the integrity and trustworthiness of the data repository, which may promote deposits of data for increased accessibility and use [31].

### Evidence-based practice

Evidence-based practice (EBP) is based on acquiring and appraising the literature. Whether it be aggregated or disaggregated data, finding appropriate literature leads to available health-related information about API communities or the lack of information on health and health care services regarding this population and subgroups. During a reference interview, a patron may ask for literature on a certain topic that may only have aggregated data. This information could be deemed as a gap in current research on the topic within API communities. The interview would give librarians an opportunity to engage with the patron around the pitfalls of aggregated data and suggest finding literature for API subgroups instead. Librarians can go beyond the reference request and provide a few additional articles or resources on available subgroup data. In addition, librarians can recommend or work on reviews about API health by gathering literature with aggregated data to highlight discrepancies of available evidence and/or collect literature on disaggregated data to provide insight on health disparities of subgroups. For example, Wyatt et al. extrapolated and synthesized aggregated and disaggregated data from existing literature to find evidence on specific risk and protective factors associated with depression and suicide among Asian American and Native Hawaiian/Pacific Islander youth [32].

### Acquiring the literature

Applying social justice principles to health sciences libraries includes keeping up with trends and engaging in critical librarianship practices [33]. Librarians need to reflect and observe societal biases and privileges within the system of information services and literature searching [33]. For example, obtaining the most relevant articles on health needs, experiences, and disparities among the API diaspora requires adequate understanding of which search terms and controlled vocabulary to use during comprehensive literature searching. For an effective search, it is important to identify and address terms and subject headings that may be problematic and/or incorrect in different literature databases. To eliminate search bias, the search strategy can be peer reviewed (by health sciences librarians and non-health sciences librarians) for appropriate use of terms and subject headings in various databases.

Knowledge of MEDLINE indexing practices can lead to better search strategies and retrieval of relevant literature [34]. Medical Subject Headings (MeSH) give uniformity and consistency to indexing and cataloging of the literature with automatic term mapping (ATM) by matching search terms to MeSH. However, there are issues with ATM, as some commonly used terms associated with the API diaspora are not appropriately matched to MeSH terms. For example, MEDLINE omits mapping of many specific Asian countries to the MeSH “Asian Continental Ancestry Group” as well as many of the diverse ethnic groups of the Pacific Islands from the MeSH “Oceanic Continental Ancestry Group.” Searchers will need to change their search strategy to reflect the lack of keywords being matched to specific MeSH terms as relevant articles may be indexed under different MeSH and pairings of subheadings [34]. For example, when searching for “Indian,” it does not map to “Asian Continental Ancestry Group” but to “American Natives.” A searcher looking for articles about the population of India or its diaspora would be required to search for “Asian Indian” or “Asian Indian American,” which maps to “Asian Americans” under “Asian Continental Ancestry Group.” In a systematic review on mental health among Asian American breast cancer survivors, the search strategy included keywords for ethnicity and country, which included “Indian” and “India” [35]. Searchers will need to be inclusive of which keywords they use for specific ethnic groups and coordinate with appropriate MeSH terms such as geographic location [34]. A systematic review on the risk of breast cancer in Asian women used geographic location subject headings with additional key terms [36]. However, searching for “South Asia” does not map to the MeSH for “Asia” but “Southern Asia” does. In addition, South Asian countries are mapped under “Western Asia,” which is geographically incorrect, and even an expert searcher could overlook this and miss relevant articles.

### Appraising the literature

EBP requires critical appraisal of research evidence to develop and implement effective clinical practice and public health programming. Librarians are often embedded in the EBP curriculum as experts in finding evidence-based resources by looking at the strengths and weaknesses of research methodology and the reliability and validity of data. One aspect of critical appraisal is applicability to a specific patient population, which is difficult to do with aggregated data.

With the evidence gap for understanding API health disparities and health care needs, librarians need to not only acquire the literature but also appraise the evidence and its applicability to specific patient populations. For example, when searching for appropriate literature, look at the tools used to collect demographic data (were they culturally appropriate?), are language barriers addressed (what languages was the survey conducted in?), inclusivity of community involvement (was the design of the study in collaboration with the specific communities it is surveying?), and/or replication of the study on different ethnic groups (has this been conducted in different API communities?) [1,2,3]. Ask these questions and instruct students, faculty, and health care professionals to think about these important elements of a research study. Focusing on disaggregated data is essential in disseminating quality evidence, especially in systematic reviews and meta-analyses as they are the highest-level of evidence (if properly conducted).

## CONCLUSION

Health sciences librarians, whether in academia or medical centers, have the unique knowledge and skill set to promote awareness around data disaggregation and advocate for its importance in understanding disparities within the API communities. Through information services, data services, EBP instruction, and patron outreach, librarians can support data disaggregation practices. Although this paper focuses on API communities, the background information on aggregated data and suggestions for librarians can be applied to other race and/or ethnic groups [3].
